# Short-term but not long-term perennial mugwort cropping increases soil organic carbon in Northern China Plain

**DOI:** 10.3389/fpls.2022.975169

**Published:** 2022-10-10

**Authors:** Zhenxing Zhou, Furong Tian, Xiang Zhao, Kunpeng Zhang, Shijie Han

**Affiliations:** ^1^ School of Biological and Food Engineering, Anyang Institute of Technology, Anyang, China; ^2^ Taihang Mountain Forest Pests Observation and Research Station of Henan Province, Linzhou, China; ^3^ International Joint Research Laboratory for Global Change Ecology, School of Life Sciences, Henan University, Kaifeng, China

**Keywords:** biomass, cropping years, enzyme activity, perennial cropping, soil organic carbon

## Abstract

Perennial cropping has been an alternative land use type due to its widely accepted role in increasing soil carbon sequestration. However, how soil organic carbon (SOC) changes and its underlying mechanisms under different cropping years are still elusive. A chronosequence (0-, 3-, 6-, 20-year) of perennial mugwort cropping was chosen to explore the SOC dynamics and the underlying mechanisms in agricultural soils of Northern China Plain. The results revealed that SOC first increased and then decreased along the 20-year chronosequence. The similar patterns were also found in soil properties (including soil ammonium nitrogen, total nitrogen and phosphorus) and two C-degrading hydrolytic enzyme activities (i.e., α-glucosidase and β-glucosidase). The path analysis demonstrated that soil ammonium nitrogen, total nitrogen, and plant biomass affected SOC primarily through the indirect impacts on soil pH, total phosphorus availability, and C-degrading hydrolytic enzyme activities. In addition, the contributions of soil properties are greater than those of biotic factors (plant biomass) to changes in SOC across the four mugwort cropping years. Nevertheless, the biotic factors may play more important roles in regulating SOC than abiotic factors in the long run. Moreover, SOC reached its maximum and was equaled to that under the conventional rotation when cropping mugwort for 7.44 and 14.88 years, respectively, which has critical implications for sustainable C sequestration of agricultural soils in Northern China Plain. Our observations suggest that short-term but not long-term perennial mugwort cropping is an alternative practice benefiting soil C sequestration and achieving the Carbon Neutrality goal in China.

## Introduction

Soils store approximately 1550 Gt organic carbon (C), dominating the global soil C pool (2500 Gt), which is 3.3 times than the atmospheric C pool (Lal, 2004). Soil organic carbon (SOC) is vital for maintaining key functions of agricultural soils, such as water and nutrient retention, microbial activity, as well as the maintenance of a physicochemical balance (Trost et al., 2013; Miller et al., 2015; Wang et al., 2019; Wiesmeier et al., 2019). Increasing SOC in agricultural soils by optimized management is a priority for achieving food security and mitigating climate change (Lal, 2004; Ledo et al., 2020; Jia et al., 2021; Beillouin et al., 2022). Previous studies have revealed that agricultural managements/land use types have substantial impacts on SOC stocks (Ledo et al., 2019; Ledo et al., 2020; Zhu et al., 2021; Kan et al., 2022; Mondal and Chakraborty, 2022). For example, proper agricultural management (such as, perennial cropping) can elevate SOC sequestration in subtropical upland soils (Zhu et al., 2021). By contrast, land use conversion from natural to agricultural ecosystems (annual or perennial crops) could lead to SOC loss (Don et al., 2011; Deng et al., 2016; Ledo et al., 2020). In fact, intensified land use changes have been observed in recent decades (Song et al., 2018; Winkler et al., 2021), however, little efforts have been made to explore the effects and underlying mechanisms of the conversion from annual to perennial crops on SOC at spatial-temporal scale.

In addition to provide food, energy feedstock, fiber, and medicinal component, perennial cropping have been also demonstrated to be an effective land use type for increasing SOC (Glover et al., 2010; Ledo et al., 2020; Zhu et al., 2021). It has been shown that conversion from natural ecosystems to perennial crops could have diverse impacts on SOC stocks. For example, land use changes from natural ecosystem to perennial crops could lead to positive (Post and Kwon, 2000; Robertson et al., 2017), negative (Don et al., 2011; Crowther et al., 2016; Deng et al., 2016), or neutral effects on SOC (Fialho and Zinn, 2012; Qin et al., 2016). The divergent impacts of perennial cropping on SOC may be attributed to different perennial crop species and soil properties. In addition, perennial cropping could result in shifts of soil physicochemical properties, such as soil C and N nutrients (Zan et al., 2001; Glover et al., 2010). These changes may have the potential to affect the growth of crops and the sustainability of soil-crop systems (Bezemer et al., 2006; Hawkers et al., 2013). Nevertheless, with the increasing of perennial cropping years, soil C sequestration may decrease due to the reduced capacity of self-sustainability (Rowe et al., 2016) or continuous cropping obstacles (Chen et al., 2020; Wang et al., 2020b; Tan et al., 2021), which pose great challenges for sustainable soil-crop systems.

In general, perennial crops have greater biomass than annual crops, providing high organic matter input into soils with crop residues, root exudates, and fine root turnover (Glover et al., 2010; Anderson-Teixeira et al., 2013; Zhu et al., 2021). Plant roots could affect SOC dynamics by root litter and rhizodeposition (Dijkstra et al., 2021). For example, SOC may gain when plant roots increase SOC stabilization through forming soil aggregates, which are less accessible to decomposition (Schimdt et al., 2011; Slessarev et al., 2020). By contrast, SOC may also lose when plant roots stimulate SOC destabilization by aggregate destruction with tillage (Hartley et al., 2012; Cheng et al., 2014; Dijkstra et al., 2021). In addition, recent evidence reveals that preference for root nitrogen uptake as ammonium nitrogen over nitrate nitrogen could lead to rhizosphere acidification, and thus inhibit the rhizosphere priming effect, with consequently promote SOC sequestration (Wang et al., 2016; Wang and Tang, 2018). Moreover, SOC could also be regulated by soil extracellular enzyme activity (Chen et al., 2018; Chen et al., 2020; Chen and Sinsabaugh, 2021). Extracellular enzymes secreted by soil microbial communities degrade complex polymers into substrates assimilating and respiring CO_2_ during the decomposition of soil organic matter (Burns et al., 2013; Bödeker et al., 2014), during which the extracellular enzymes play a critical role during the decomposition of soil organic matter (Sinsabaugh et al., 2008; Cenini et al., 2016). Due to less soil disturbance associated with lower tillage, conversion from annual to perennial cropping may have the potential to increase soil microbial enzyme activities (Haney et al., 2010; Dou et al., 2013). In addition, soil chemical properties under less soil disturbance can provide steady nutrient supply for extracellular enzymes (Cattaneo et al., 2014; Zhang et al., 2021a). Thus, conversion from annual to perennial cropping may have substantial impacts on SOC through affecting enzyme activities. Nevertheless, the relative contributions of diverse soil and plant properties to SOC under the conversion from annual to perennial cropping remain unclear.

Considering that perennial crops dominate 30% of global croplands (Ledo et al., 2018) and usually have greater biomass than conventional crops, perennial cropping has the substantial potential to sequester more C into agricultural soils and thus mitigate climate change (Ledo et al., 2019; Ledo et al., 2020). As a perennial crop, mugwort (*Artemisia argyi* Lévl. et Van) has been widely cultivated in recent years due to its medicinal value in Northern China Plain (Jiang et al., 2019). In this study, a chronosequence (0, 3, 6, and 20 years) of perennial mugwort cropping was identified and chosen in Northern China Plain. Soil and climate conditions were similar among the four sampling sites, which provided us the opportunity for using the space-for-time substitution method to explore the scientific questions: 1) what are SOC dynamics along the chronosequences of mugwort cropping? 2) which factors drive the changes in SOC under different cropping years of mugwort?

## Materials and methods

### Site description and sample collection

This study was conducted in Tangyin County (35°45′-36°01′ N, 114°13′-114°42′ E), Anyang, Henan, where is one of the origins of mugwort, a genuine herb. This region has a warm temperate continental monsoon climate, with mean annual temperature of 13.4°C and precipitation of 582.0 mm, respectively. The soil is classified as cinnamon soil according to the Chinese soil classification system. The surface soil contained organic matter of 16.7 mg/g and total nitrogen content of 1.07 mg/g. With the development of the traditional Chinese medicine industry, parts of croplands were gradually converted from maize-wheat rotation to perennial mugwort cropping in Tangyin County in recent years, which provides the opportunity to assess the effects of the chronosequence of perennial mugwort cropping on soil organic carbon.

We identified and selected a chronosequence (continuous maize-wheat rotation (Control-Y0), perennial mugwort cropping for 3 (Y3), 6(Y6), and 20 years(Y20)) of perennial mugwort cropping in this study region. Given the distances among the four chronosequence periods were close (less than 5 km), we considered that soil types among the four chronosequence periods were not heterogeneous. In each chronosequence period, three blocks were randomly established. The aboveground plant parts of each plot in each block were harvested and put into tagged mash bags in late September of 2020. Then, three 20 cm depth (0-10 cm, 10-20 cm) cylindrical holes were excavated using a soil auger (5 cm in diameter) in the plots. Soil samples were passed through a 0.25 mm sieve and roots were collected then oven-dried at 105°C for 48 hours. Then, soil samples were divided into two subsamples. One subsample was stored at 4°C for measuring soil physicochemical properties. Another subsample was stored at -20°C for measuring soil extracellular enzymatic activities (α- (AG) and β-glucosidase (BG)).

### Soil chemical properties and extracellular enzyme activities

Soil organic carbon and total N content were measured by an elemental analyzer with a dry combustion method (Vario MAX CN, Elementar Co., Germany). The total phosphorus (P) content was determined by H_2_SO_4_-HClO_4_ digestion and then P molybdenum blue colorimetric analysis (Murphy and Riley, 1962). The concentrations of ammonium (NH_4_^+^) and nitrate-nitrogen (NO_3_^-^) were extracted with 2M KCl solution and measured by a flow injection analyzer (SAN-System, Netherlands). Soil pH was measured with a combination glass-electrode [soil:water = 1:2.5 (W/V)] (Tian et al., 2022).

α-glucosidase (AG) and β-glucosidase (BG) are two key C-degrading hydrolytic enzymes. The activities of the two enzymes were determined by a colorimetric method described previously using fluorescently-labeled substrates (German et al., 2011; Allison et al., 2018). Fresh soil sample (0.2 g dry weight) was homogenized in 100 mL 25 mM maleate buffer (pH = 6.0). 125 µL of fluorometric substrate solution in 25 mM maleate buffer was mixed with 125 µL soil homogenate in each well of a 96-well microplate. Then, we incubated the 96-well microplate for 4 hours for analyzing the two hydrolytic enzymes (AG and BG). Fluorescent signals for the two enzymes were acquired at 365 nm excitation and 450 nm emission (BioTek Synergy H1 microplate reader, Winooski, VT, USA). The enzyme activity was expressed as nmol h^-1^ g^-1^ dry soil based on the method described by German et al. (2011).

### Statistical analyses

All data are presented as mean values ± standard deviation (SD) for the three plots in each cropping type. Two-way ANOVAs were used to explore the effects of soil depth and cropping year on all the variables included in the study. In addition, changes in soil chemical properties among the four cropping types were assessed using one-way ANOVA with Duncan multiple comparisons. After that, regression analyses were conducted between SOC and cropping years to fit the SOC dynamic. The correlations among variables were explored by the Pearson correlation method. Significant differences were evaluated at the 0.05 probability level.

Random Forest (RF) models were used to partition independent influences of NH_4_^+^, NO_3_^-^, NH_4_^+^/NO_3_^-^, TN, TP, pH, AG, BG, root biomass (RB), and aboveground biomass (AGB) on SOC. Then, path analysis was employed to explore a mechanistic understanding of the direct and indirect effects of soil properties and enzyme activities, as well as RB and AGB on SOC along the chronosequence of perennial mugwort cropping. We first examined whether there were any collinear relationships between the factors by calculating the variance inflation factor (VIF). The factors were excluded if VIF was more than 5. Due to there were no significant relationships of SOC with NO_3_^-^ and RB/AGB, or weak relationships between SOC and NH_4_^+^/NO_3_^-^ in linear regression analyses, NO_3_^-^, RB/AGB, and NH_4_^+^/NO_3_^-^ were not included in the path analysis. The path analysis was achieved based on the maximum likelihood method, Chi-square (χ^2^), degree of freedom (df), root-mean-squared error (RMSE), AIC value, and goodness of fit index (GFI). The path analysis was performed using AMOS 20.0 (AMOS Development Corporation, Chicago, IL, USA) and other statistical analyses were performed using SAS 8.0 (SAS Institute Inc., Cary, NC, USA) and R v.4.1.1 (R Development Core Team). Excel 2019 (Microsoft Crop., Redmond, WA, USA) and GraphPad Prism 9.0 software (GraphPad Inc., San Diego, California, USA) were used to plot the graphs.

## Results

### Soil properties

**Figure 1:**
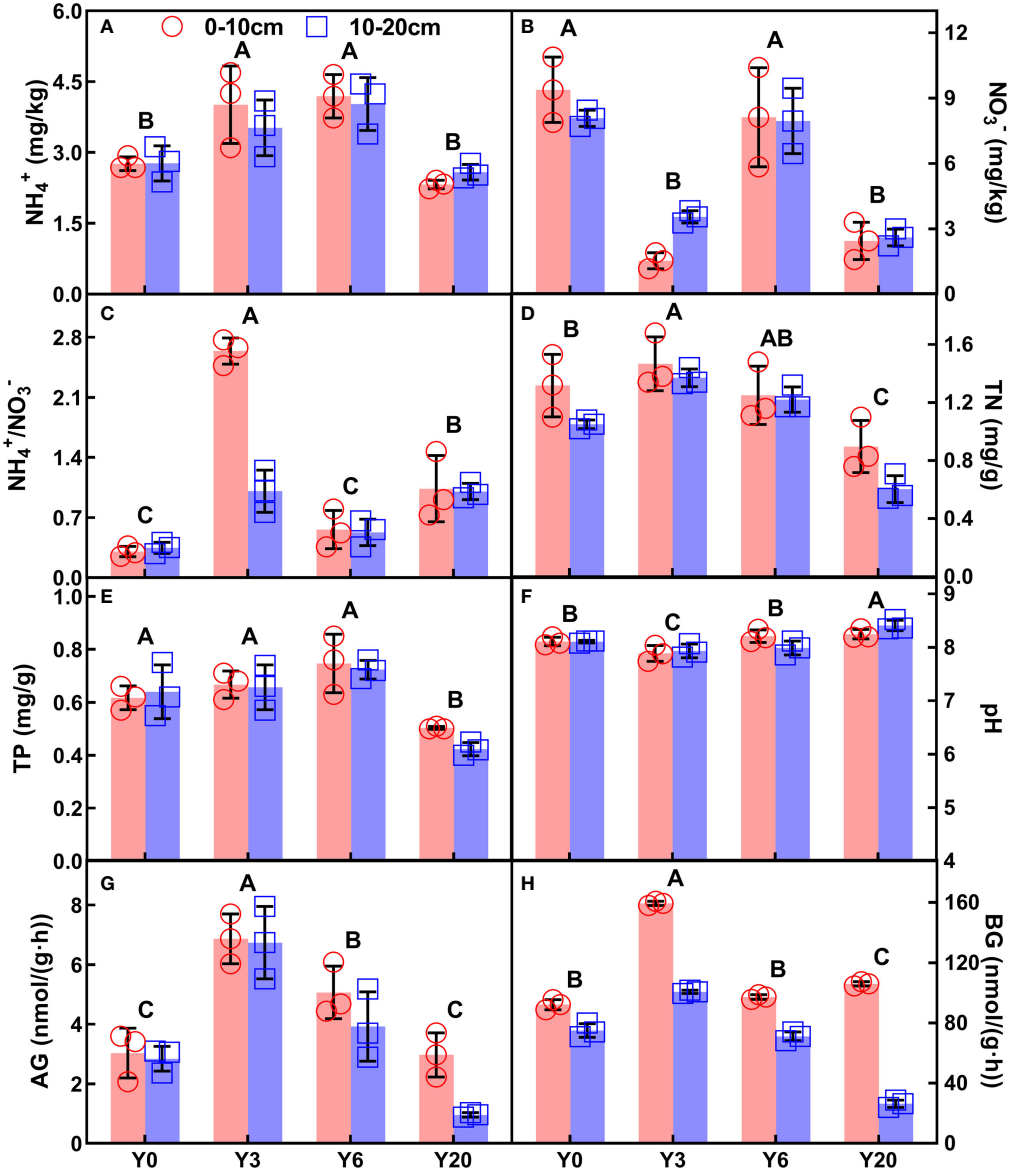
NH_4_^+^ **(A)**, NO_3_^-^ **(B)**, the ration of NH_4_^+^ to NO_3_^-^ (NH_4_^+^/NO_3_^-^, **C**), TN **(D)**, TP **(E)**, pH **(F)**, AG **(G)**, and BG **(H)** (Mean ±SD) of different soil depths (0-10 cm and 10-20 cm) along the chronosequence of mugwort cropping. Y0: continuous maize-wheat rotation; Y3: mugwort cropping for 3 years (since 2017); Y6: mugwort cropping for 6 years (since 2014); Y20: mugwort cropping for 20 years. Different letters indicate significant differences among the four cropping years at *P* < 0.05. See **Table 1** for abbreviations.

**Figure 2:**
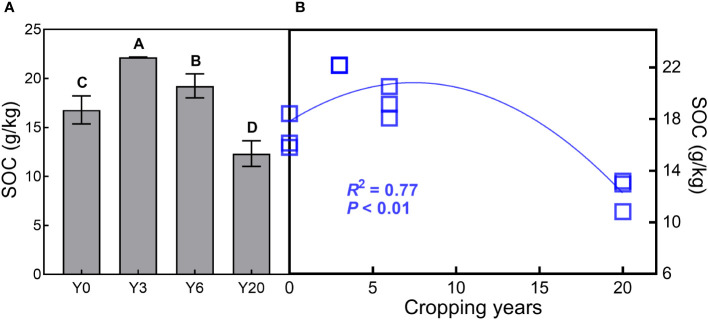
Soil organic carbon content across the two soil depths (SOC, g/kg, n=3, Mean ± SD, **A)**, and the relationship of SOC with cropping years **(B)**. Different letters indicate significant difference among the four cropping years at *P* < 0.05. See **Figure 1** for abbreviations.

Soil ammonium nitrogen (NH_4_^+^), nitrate-nitrogen (NO_3_^-^), NH_4_^+^/NO_3_^-^, total N and phosphorus (P), pH, α-glucosidase (AG) and β-glucosidase (BG), as well as SOC showed significant variations among different mugwort cropping years across the two depths (**Figures 1**, **2**, all *P* < 0.001, **Table 1**). NH_4_^+^, TN, TP, AG, BG, and SOC showed a trend of first increase and then decrease along the chronosequence (Y0-Y20, **Figures 1A, D, E, G, H**, **2A, B**). TN and TP, BG, as well as SOC were lowest in 20-year mugwort cropping soils (**Figures 1D, E, H**). In addition, the highest of NH_4_^+^/NO_3_^-^, TN, AG, BG, and SOC were found in the 3-year mugwort cropping soils (**Figures 1C, D, G, H**). When simulating the SOC against with year (*R* ^2^ = 0.77, *P* < 0.001, **Figure 2B**), we found that SOC reached its maximum (20.82 g/kg) in the year of 7.44 and was equal to that under the conventional rotation when cropping mugwort for 14.88 years (**Figure S1**).

**Table 1:** Effects of soil depth (Depth) and cropping year (Year) on soil ammonium-nitrogen content (NH_4_^+^), nitrate-nitrogen content (NO_3_^-^), the ratio of NH_4_^+^ to NO_3_^-^ (NH_4_^+^/NO_3_^-^), total nitrogen content (TN), total phosphorus content (TP), pH, two C-acquiring enzymes: α- (AG) and β-glucosidase (BG), and soil organic carbon content (SOC).

Variations	Depth		Year		Depth×Year	
	F	P	F	P	F	P
NH_4_^+^	0.26	0.62	17.16	<0.001	0.68	0.58
NO_3_^-^	0.14	0.72	50.42	<0.001	2.09	0.14
NH_4_^+^/NO_3_^-^	25.53	<0.001	65.57	<0.001	24.63	<0.001
TN	8.13	0.01	22.09	<0.001	1.14	0.36
TP	0.67	0.43	17.42	<0.001	0.61	0.62
pH	0.01	0.94	15.04	<0.001	3.31	0.05
AG	6.33	0.02	37.31	<0.001	1.67	0.21
BG	1828.52	<0.001	661.95	<0.001	186.44	<0.001
SOC	18.11	<0.001	59.76	<0.001	9.20	<0.001

The bold numerals indicate the significance at *P* ≤ 0.05.

### Plant properties

The lowest root biomass was observed in 0-year mugwort cropping (**Table 2**). There was no difference in root biomass among the 3-year, 6-year, or 20-year mugwort cropping. Aboveground biomass showed a decreasing trend along the chronosequence of mugwort cropping (**Table 2**). The ratio of RB to AGB (RB/AGB) in 0-year mugwort cropping soils was lower than that in the other three chronosequence cropping periods. No differences in RB/AGB were detected among the 3-year, 6-year, or 20-year mugwort cropping (**Table 2**).

**Table 2:** Mean values (Mean ± 1SE) of root biomass (RB), aboveground biomass (AGB), and the ratio of RB to AGB (RB/AGB) across the plots along the chronosequence of mugwort cropping.

Cropping years	RB (kg/m^2^)	AGB (kg/m^2^)	RB/AGB
Y0	0.15 ± 0.06b	1.43 ± 0.05a	0.11 ± 0.04b
Y3	0.77 ± 0.15a	1.08 ± 0.04b	0.71 ± 0.14a
Y6	0.71 ± 0.25a	0.89 ± 0.01c	0.80 ± 0.29a
Y20	0.51 ± 0.22a	0.69 ± 0.05d	0.77 ± 0.37a

Different letters indicate significant differences among the four cropping years.

### Relationships among soil and plant properties

**Figure 3:**
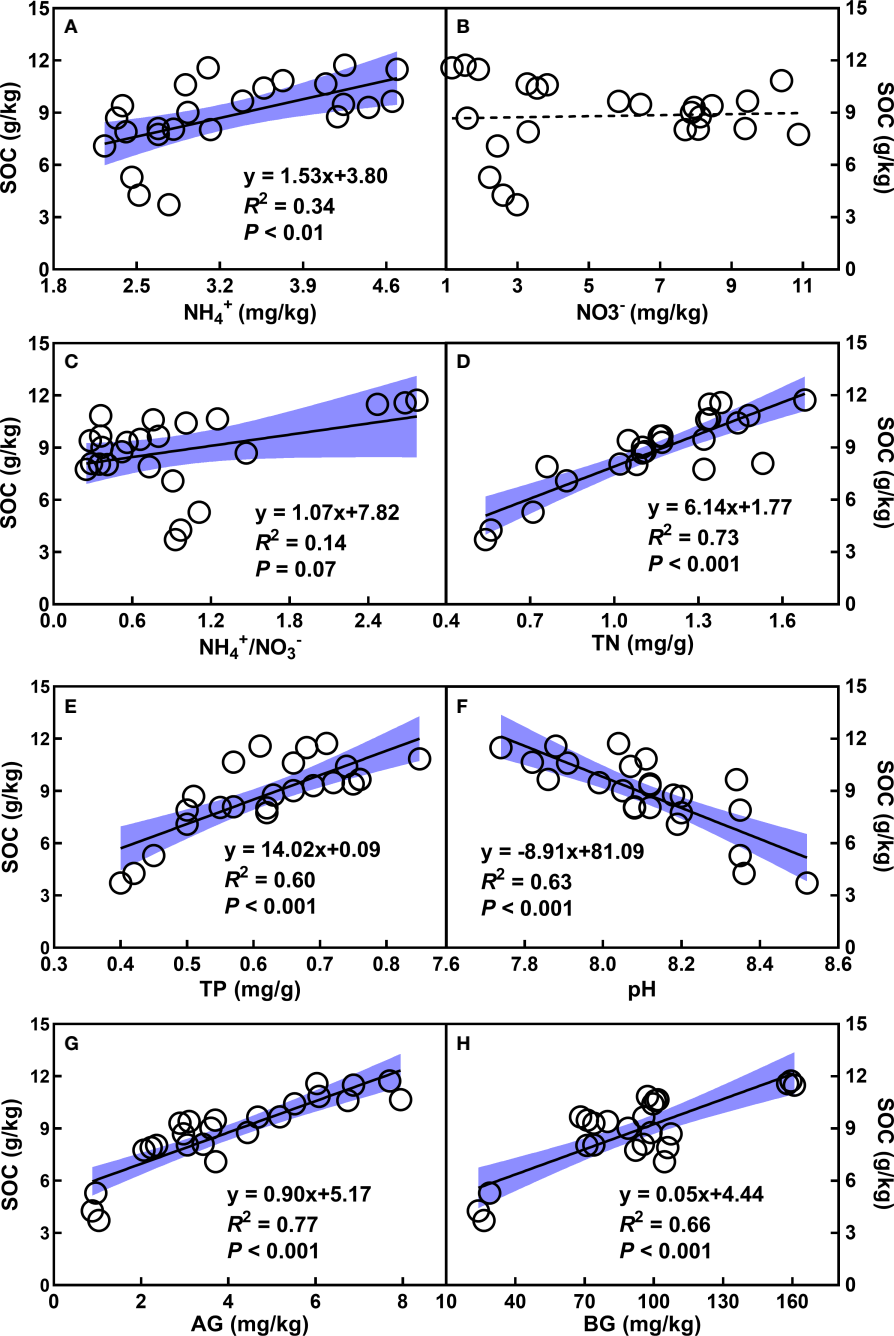
Relationships of SOC (g/kg) with NH_4_^+^ **(A)**, NO_3_^-^ **(B)**, the ratio of NH_4_^+^ to NO_3_^-^ **(C)**, TN **(D)**, TP **(E)**, pH **(F)**, AG **(G)**, and BG **(H)**, respectively. The shadow areas represent 95% confidence intervals. See **Table 1** for abbreviations.

**Figure 4:**
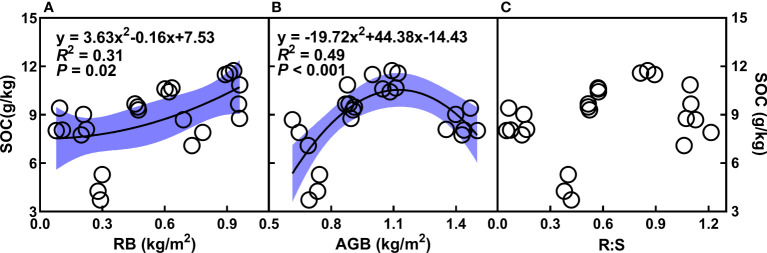
Relationships of SOC (g/kg) with root (RB, kg/m^2^, **A**) and aboveground biomass (AGB, g/m^2^, **B**), as well as the ratio of root biomass to aboveground biomass (RB/AGB, **C**) under the four cropping durations. The shadow areas represent 95% confidence intervals.

**Figure 5:**
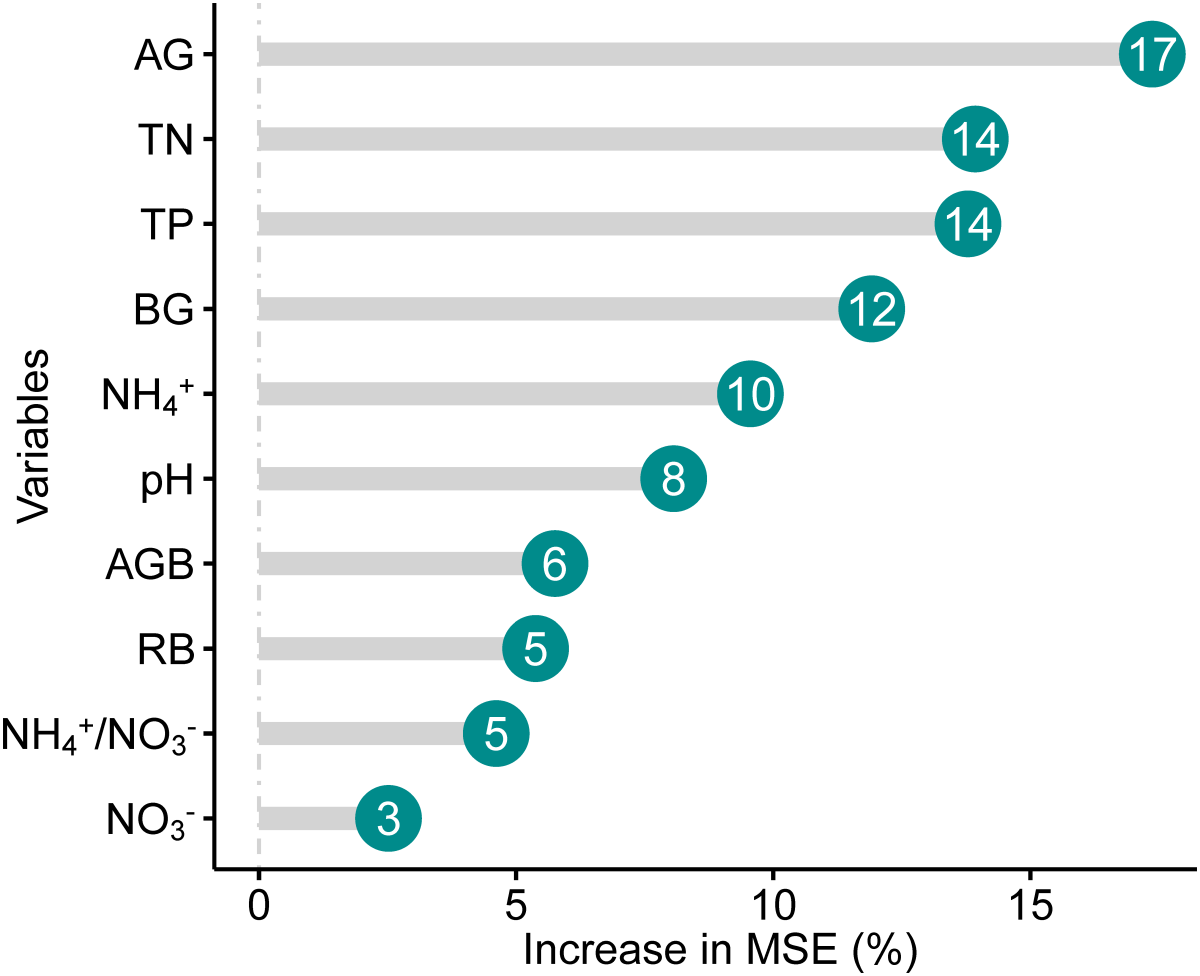
Relative contributions (Increase in MSE, %) of different factors to SOC. See **Table 1** for abbreviations.

**Figure 6:**
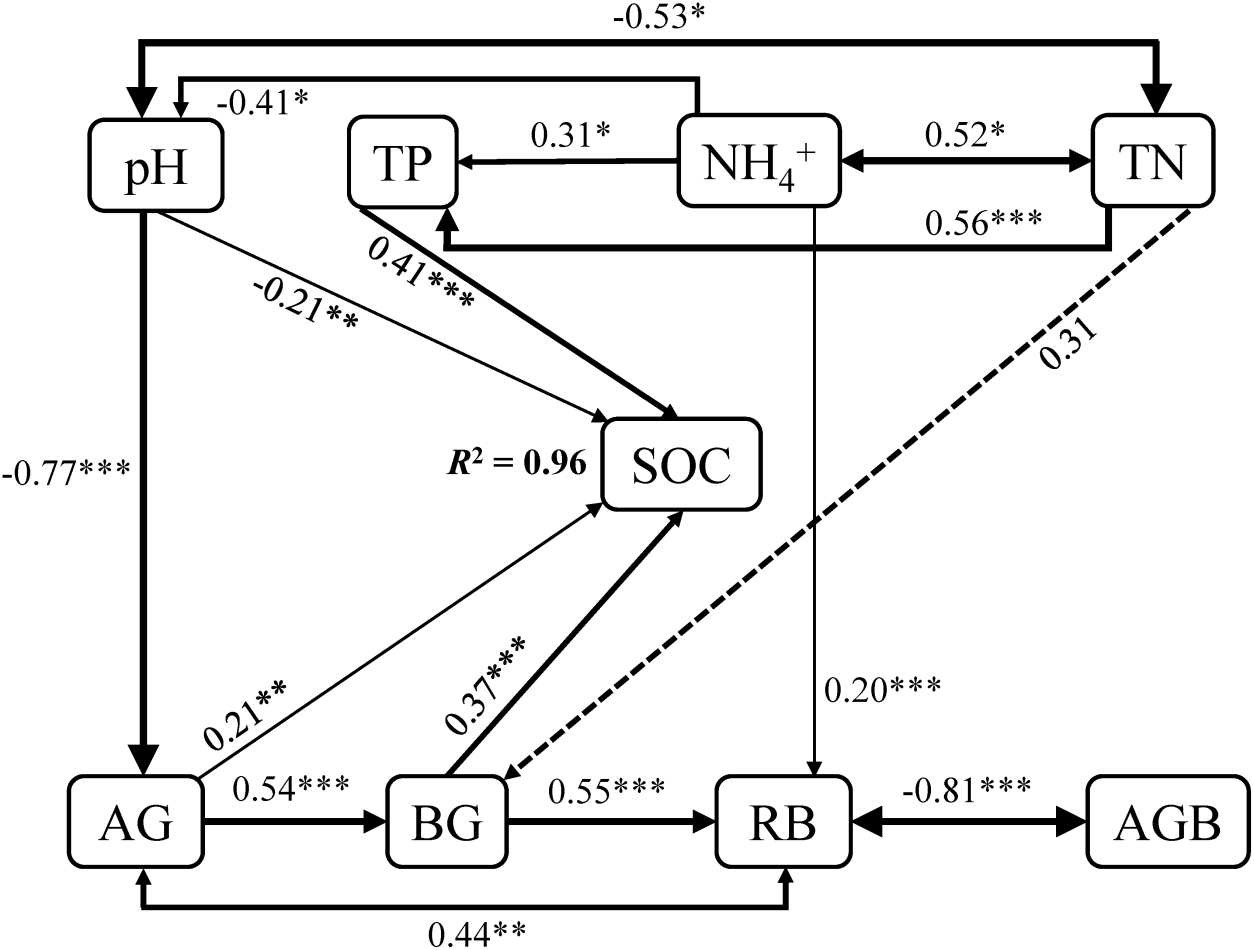
Path analysis showing the effects of pH, TP, NH_4_^+^, TN, AG, BG, as well as RB and AGB on SOC. The final path analysis adequately fitted the data χ^2^ = 28.55, df = 20, *P* = 0.10, GFI = 0.83, AIC = 78.55, RSME = 0.001. Solid and dashed arrows suggest significant and non-significant paths, respectively. The width of the solid arrows indicates the strength of the relationships. *R* ^2^ shows the proportion of SOC explained by variables. Significant level: * *P* < 0.05; ** *P* < 0.01; *** *P* < 0.001.

Soil organic carbon (SOC) enhanced with increasing NH_4_^+^, NH_4_^+^/NO_3_^-^, TN, TP, as well as AG and BG (**Figures 3A, C–E, G, H**), whereas decreased with the augment of pH (**Figures 3F, G**). In addition, the quadratic relationships of SOC with root and aboveground biomass were observed (**Figures 4A, B**). There was no relationship of SOC with RB/AGB (**Figure 4C**). The RF models showed that AG and BG accounted for 17.59% and 10.93% of SOC variance, respectively (**Figure 5**). Path diagrams showed interdependence relationships of TN with NH_4_^+^ (*r* = 0.52, *P* = 0.03) and pH (*r* = -0.53, *P* = 0.01), as well as of RB with AG (*r* = 0.44, *P* < 0.01) and AGB (*r* = -0.81, *P* < 0.01), respectively (**Figure 6**).

In addition, neither AGB nor RB had effects on SOC directly, whereas affect SOC by altering AG and BG indirectly. Changes in RB had positive impacts on AG (*r* = 0.44, *P* < 0.01), with consequently alter the SOC (*r* = 0.21, *P* < 0.01). Moreover, changes in NH_4_^+^ have impacts on SOC mainly through affecting pH (*r* = -0.41, *P* = 0.03; *r* = -0.21, *P* < 0.01) and TP (*r* = 0.31, *P* = 0.05; *r* = 0.41, *P* < 0.001). RB increased with increments of NH_4_^+^, indicating that NH_4_^+^ could also mediate SOC through altering RB (*r* = 0.20, *P* < 0.001), and thus AG (**Figure 6**). Furthermore, the interdependence relationship of TN with pH, as well as the positive dependence of TP on TN, suggesting that changes in TN had noticeable influences on SOC by altering TP (*r* = 0.56, *P* < 0.001; *r* = 0.41, *P* < 0.001) and pH (*r* = -0.53, *P* = 0.01; *r* = -0.21, *P* < 0.01). Given that the positive relationship of BG with AG (*r* = 0.54, *P* < 0.001), variables that affect AG could thus alter BG, and consequently change SOC (**Figure 6**).

## Discussion

### Changes in SOC along the chronosequence of perennial mugwort cropping

In this study, SOC under 3- and 6-year cropping of perennial mugwort is greater than that under conventional rotation, which indicates that short-term perennial mugwort could be an effective practice for improving soil C sequestration. This finding is consistent with those reported by previous studies, which have revealed that perennial cropping can have fundamental positive impacts on soil C sequestration (Ledo et al., 2019; Ledo et al., 2020; Zhu et al., 2021). However, mugwort cropping begins to decrease SOC after 7.44 years (especially after 14.88 years: SOC is lower than that under the conventional rotation), suggesting that long-term perennial mugwort cropping could not benefit for soil C sequestration, which may lead to positive feedbacks to climate change (Lal, 2004; Ledo et al., 2020; Jia et al., 2021).

When analyzed by different soil depths, SOC under mugwort cropping at the 20-year chronosequence was equivalent to that under maize cropping (Y0) at the depth of 10 cm (**Figure S1**). Therefore, the observations of reduced SOC across the two depths can be largely attributed to decreased SOC at the depth of 20 cm (**Figure S1**), indicating that subsoil SOC may decrease more quickly than topsoil SOC under mugwort cropping at the long-term scale. The findings that decreased patterns of SOC with soil depth observed in this study are consistent with those reported in previous studies (Rentschler et al., 2019; Zhu et al., 2021). The above observations contribute to the decreased SOC across the two depths over the chronosequence of mugwort cropping. The trends reported in this study make it reasonably clear that short-term mugwort cropping is beneficial to SOC sequestration, whereas long-term (more than 14.88 years) mugwort cropping has the reverse impacts on SOC sequestration, indicating that mugwort should be rotated with other crops to prevent the decrease of SOC compared with that under conventional crops. However, recent evidence reveals that SOC storage of deep soil may show change compared with that of surface soil under perennial cropping (Ledo et al., 2020). For example, conversion from annual to perennial crops can result in an average 20% enhancement in SOC at 0-30 cm and 10% increase across the 0-100 cm soil profile (Ledo et al., 2020). Therefore, accurate evaluation on the dynamics of SOC under perennial cropping needs to consider deeper profiles (such as, up to 60 cm or 100 cm depth) in future studies (Ledo et al., 2020; Kan et al., 2022; Chen et al., 2022). Nevertheless, lack of cropping chronosequence between 6 and 20 years, during which there may be uncertainties in SOC shifts. Therefore, more cropping years are needed to be considered in future field studies.

### Changes in aboveground and root biomass and their impacts on SOC

Aboveground and root biomass play important roles in regulating SOC through providing C input (Anderson-Teixeira et al., 2013; Waring et al., 2014; Deng et al., 2019; Zhou et al., 2020). In this study, the aboveground biomass of mugwort was lower than that of maize, whereas root biomass of mugwort was greater than that of maize (**Table 2**). Under the short-term mugwort cropping, higher root biomass could stimulate root turnover and C exudation, and thus increase SOC (Beare et al., 2014). Under the long-term (more than 14 years) mugwort cropping, although root biomass of mugwort is still greater compared with that of maize, the turnover of mugwort root may be lower than that of maize root due to continuous cropping obstacle, which is demonstrated in other crops (Chen et al., 2020; Wang et al., 2020b; Tan et al., 2021), leading to reduced root exudation (Leifeld et al., 2015; McNally et al., 2017), and consequently result in lower SOC. In addition, decreased aboveground biomass under perennial mugwort cropping, leading to a reduction of crop C input into soil, which may further depress SOC at a long-term scale (Cai et al., 2022; Krauss et al., 2022).

### Changes in soil properties and their impacts on SOC

The finding of short-term (Y3 and Y6) consecutive mugwort cropping increased NH_4_^+^, total N and P of surface soil (**Figures 1A, D, E**, **2A**) are consistent with that reported in a previous study (Zhu et al., 2021). In the current study, although short-term mugwort cropping decreased aboveground biomass, increased root biomass combining the enhanced soil NH_4_^+^, total N and P nutrient could stimulate the turnover rate of roots (Sun et al., 2017), which is beneficial to sequestrate C into the soil. The observations of positive and quadratic relationships of SOC with root biomass and aboveground biomass, respectively (**Figures 4A, B**) support the above discussion. In addition, less tillage associated with conversion from annual crops (Y0) to perennial mugwort could decrease the fragmentation of soil aggregates and thus decomposition of soil organic matters (Liu et al., 2021b; Kan et al., 2022; Lessmann et al., 2022; Mondal and Chakraborty, 2022), which can increase the storage of SOC. Moreover, it has been documented that enzyme activity plays a vital role in regulating organic carbon in agricultural soils and shows a positive relationship with SOC (Cattaneo et al., 2014; Zhang et al., 2021a). The observations of positive dependences of SOC with AG and BG in this study are consistent with those found in previous studies (Cattaneo et al., 2014; Zhang et al., 2021a). In this study, increased root biomass could stimulate root turnover and associated substrate accumulation (e.g., dead root, root exudation, etc.). The extent of increased substrate accumulation may exceed that decomposed by enzymes, and thus resulting in the positive relationships between SOC and enzyme activities, with consequently increase SOC. Furthermore, decreased pH may inhibit soil respiration and thus the C mineralization process (Kemmitt et al., 2006; Lazicki et al., 2022), with consequently contribute to the increased SOC under the short-term mugwort cropping. Less soil disturbance under consecutive mugwort cropping with lower tillage may also protect the aggregates from fragmentation and thus the loss of SOC (Zhu et al., 2014; Lal, 2015; Liu et al., 2021b; Kan et al., 2022; Mondal and Chakraborty, 2022). As a consequence, consecutive perennial mugwort cropping could increase SOC sequestration at the short-term scale in the temperate regions of Northern China Plain.

By contrast, at the long-term scale (20-year mugwort cropping), changes in soil properties (soil NH_4_^+^, total N and P, as well as AG and BG) were reversed compared with those at the short-term scale (**Figure 1**). The decreased above soil properties (soil NH_4_^+^, total N and P) may be ascribed to two reasons. First, long-term monoculture could lead to intensified intraspecific competition and continuous cropping obstacles (Chen et al., 2020; Wang et al., 2020b; Liu et al., 2021a; Tan et al., 2021), and thus result in lower nutrient use efficiency, which depress the aboveground biomass (**Table 2**), further decrease the transfer of C from plant to the soil (Wang et al., 2020b), with consequently inhibit the enzyme activities (i.e., AG and BG, **Figures 1G, H**) and soil nutrient availability. Second, decreased soil total N and P associated with reduced nutrient use efficiency may result from the leaching effects, and exacerbate the positive feedbacks of aboveground biomass reduction. Decreased soil total N could lead to lower soil C/N, which further reduce the stability and resistance to the decomposition of soil organic matters, resulting in decreased SOC (McNally et al., 2017; Wang and Tang, 2018; Ni et al., 2022). Regression and path analyses showed that there were indirect impacts of aboveground biomass on SOC through affecting root biomass and thus AG and BG activities (**Figures 4**, **6**). In addition, given the important role of pH in regulating C mineralization (Kemmitt et al., 2006; Zhang et al., 2021b), increased pH associated with decreased soil total N could stimulate soil respiration (C mineralization process) and consequently reduce SOC under long-term mugwort cropping (**Figures 1**, **3**, **6**), which is consistent with that found in a recent study (Lu et al., 2022). Irrespective of the temporal impacts of mugwort cropping on SOC, the contributions of C-degrading hydrolytic enzyme activities (i.e., AG and BG) and soil nutrients (e.g., TN and TP, **Figure 5**) were greater than those of biotic factors (aboveground and root biomass) on SOC along the chronosequence cropping. These observations indicate that soil properties play more important roles than biotic factors in regulating SOC at the spatial scale in agricultural soils, which is also supported by the path analysis (**Figure 6**). It has been demonstrated that long-term conservation tillage (such as, no-tillage) could increase the abundances of plant pathogens, resulting in root rot and thus reduction of plant biomass (Wang et al., 2020a), with consequently further decrease the substrate supply for accumulation of SOC in the long run. As a consequence, the importance of biotic factors may exceed than that of abiotic factors under the long-term mugwort cropping. Overall, our study has critical implications for sustainable mugwort cropping and mitigation of climate change. The findings of first increased and then decreased SOC along the chronosequence of mugwort cropping suggest that short-term (less than 7.44 years) mugwort cropping could be beneficial for maximized C sequestration, whereas long-term mugwort cropping has a negative impact on SOC. These observations indicate that long-term mugwort cropping (more than 14 years) should be avoided to access the goals of peak C emission and neutrality (Wang et al., 2021).

### Conclusions

Using a data set of SOC along a chronosequence of perennial mugwort cropping in Northern China Plain, we demonstrated that SOC increased under the short-term cropping and reached the maximum in the year of 7.44, whereas decreased under the long-term cropping (more than 14.88 years). Soil properties and C-degrading hydrolytic enzyme activities contributed more than biotic factors (aboveground and root biomass) in regulating SOC across the chronosequence. Nevertheless, the role of biotic factors may exceed that of abiotic factors in mediating SOC under long-term mugwort cropping. Given its economic benefit and critical role in regulating SOC sequestration, short-term (less than 7 years) perennial mugwort cropping is an alternative practice to maximumly sequestrate more C into the soil in Northern China Plain. Our findings that contrast impacts of short-term and long-term perennial mugwort cropping on SOC suggest that long-term perennial mugwort cropping may not beneficial for maintaining soil C storage and achieving the goals of C neutrality of China.

## Data availability statement

The original contributions presented in the study are included in the article/**Supplementary Material**. Further inquiries can be directed to the corresponding authors.

## Author contributions

ZZ originally formulated the idea, ZZ, FT, and XZ developed methodology, ZZ and FT conducted fieldwork, ZZ and FT generated data analyses, ZZ, KZ, and SH wrote the manuscript. All authors contributed to the article and approved the submitted version.

## Funding

This work was financially supported by the Postdoctoral Innovation and Practice Base of Anyang Institute of Technology (BSJ2020021, BHJ2021007). This work was financially supported by the Postdoctoral Innovation and Practice Base of Anyang Institute of Technology (BSJ2020021, BHJ2021007) and the National Natural Science Foundation of China (32201366).

## Acknowledgments

The authors thank Qingfeng Li and Mingdong Chen for their help in the field sampling, Prof. Guoyong Li for his suggestions for improving this manuscript.

## Conflict of interest

The authors declare that the research was conducted in the absence of any commercial or financial relationships that could be construed as a potential conflict of interest.

## Publisher’s note

All claims expressed in this article are solely those of the authors and do not necessarily represent those of their affiliated organizations, or those of the publisher, the editors and the reviewers. Any product that may be evaluated in this article, or claim that may be made by its manufacturer, is not guaranteed or endorsed by the publisher.
