# Supplementary material for: Short-term but not long-term perennial mugwort cropping increases soil organic carbon in Northern China Plain
Source: Front Plant Sci. 2022 Oct 10;13:975169. doi: 10.3389/fpls.2022.975169 (PMC9589220; doi:10.3389/fpls.2022.975169)

**Figure S1.** Soil organic carbon content in the two soil depths (0-10 cm and 10-20 cm, n=3, Mean ± SD). The curves represent the relationships of SOC with cropping years in the 0-10 cm and 10-20 cm, respectively.

**Figure S2.** Correlations among soil and plant properties in this study. See abbreviations in Table 1.


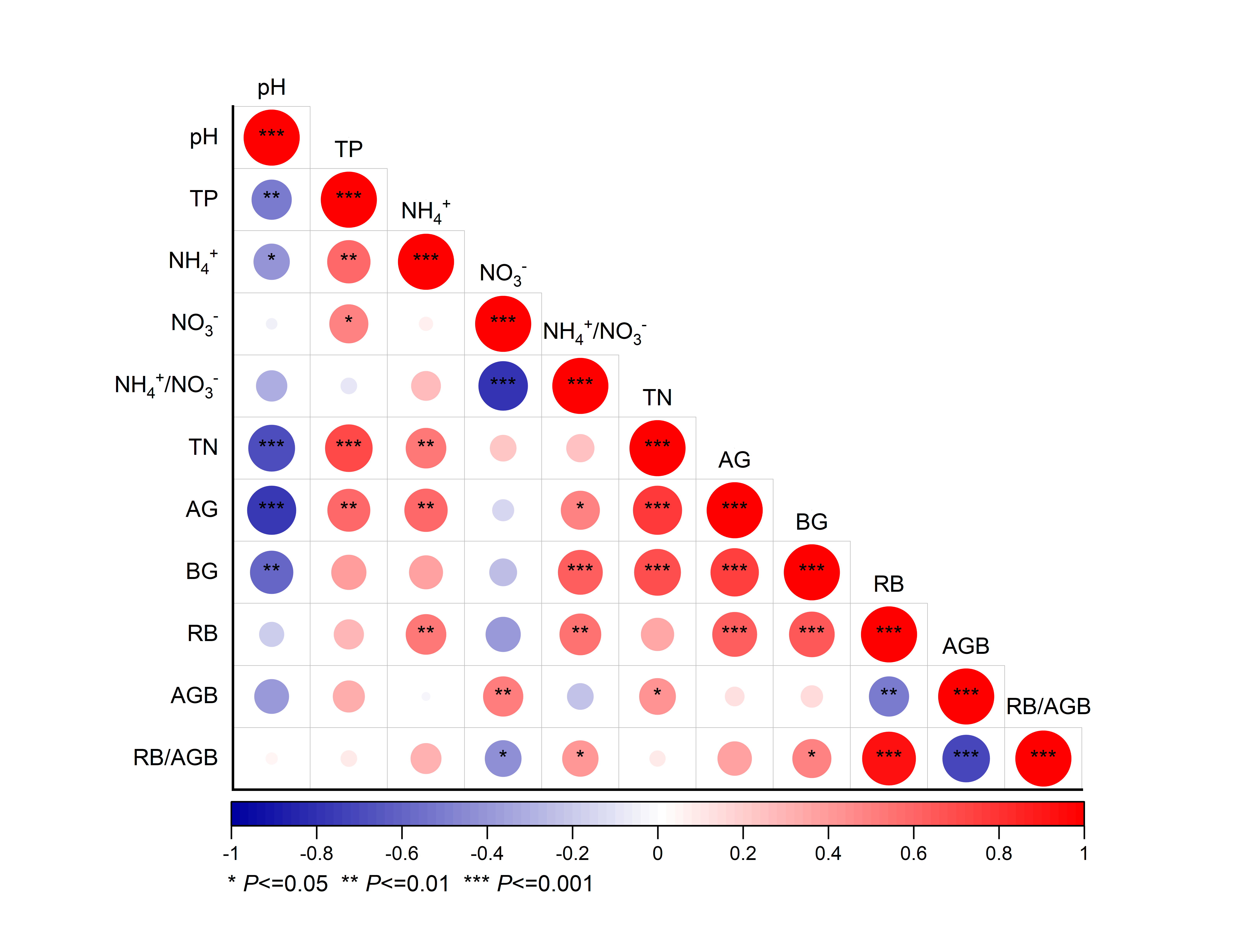

Supplement: Supplementary file 1 [file DataSheet_1.docx]
